# Investigation of proteomic profiles in canine lymphoma using tandem mass tag-based quantitative proteomics approach

**DOI:** 10.14202/vetworld.2022.1333-1340

**Published:** 2022-05-26

**Authors:** Piyanoot Fonghem, Trairak Pisitkun, Kasem Rattanapinyopituk, Sirintra Sirivisoot, Anudep Rungsipipat

**Affiliations:** 1 Center of Excellence for Companion Animal Cancer, Department of Veterinary Pathology, Faculty of Veterinary Science, Chulalongkorn University, Bangkok, Thailand; 2 Center of Excellence in Systems Biology, Faculty of Medicine, Chulalongkorn University, Bangkok, Thailand

**Keywords:** B-cell lymphoma, dog, tandem mass tag proteomics, T-cell lymphoma

## Abstract

**Background and Aim:**

Specific tumor biomarkers are useful for the early diagnosis of cancer or can predict the recurrence of neoplastic disease in humans and animals. Lymphoma in dogs could be classified into B-, T-, and NK-cell origins. T-cell lymphoma has the worst prognosis with a shorter survival time and disease-free interval. This study aimed to identify the differential serum protein expressions of canine B- and T-cell lymphomas compared with healthy dogs using a tandem mass tag (TMT)-based quantitative proteomics.

**Materials and Methods:**

Serum samples were collected from 20 untreated canine lymphomas (14 B-cells and 6 T-cells) and four healthy control dogs. Sera peptides from each sample were processed for TMT 10-plex tagging and analyzed using liquid chromatography-mass spectrometry (MS). Differential proteome profiling was then compared between lymphoma and control.

**Results:**

We discovered 20 elevated and 14 decreased serum proteins in the lymphoma group relative to the healthy group. Six candidate increased proteins in canine lymphomas were beta-actin cytoplasmic 1 (ACTB, p=0.04), haptoglobin (p=0.002), beta-2 microglobulin (aaaaaaaa2M, p=0.007), beta-2 glycoprotein 1 (APOH, p=0.03), metalloproteinase inhibitor 1 (TIMP-1, p=0.03), and CD44 antigen (p=0.02). When compared between B- and T-cell lymphomas, B-cell phenotypes had upregulated immunoglobulin (Ig) heavy chain V region GOM (p=0.02), clusterin (p=0.01), apolipoprotein C1 (APOC1, p=0.05), and plasminogen (p=0.02).

**Conclusion:**

These findings were investigated quantitative serum proteomes between B- and T-cell lymphomas using TMT-based MS. ACTB, aaaaaaaa2M, APOH, TIMP-1, CD44 antigen, Ig heavy chain V region GOM, and APOC1 are novel candidate proteins and might serve as a lymphoma biomarker in dogs. However, evaluation with an increased sample size is needed to confirm their diagnostic and prognostic ability.

## Introduction

Canine lymphoma is the most common hematopoietic cancer and multicentric lymphoma is frequently observed in dogs [1]. Based on the World Health Organization (WHO) classification, lymphoma can be subtyped into B-cell, T-cell, and non-B non-T-cell lymphomas [2]. Dogs with high-grade T-cell lymphoma tend to have decreased response rate, progression-free survival, and overall survival time when compared to B-cell lymphoma treated with the same CHOP (vincristine, cyclophosphamide, doxorubicin and prednisolone)-based chemotherapy [3]. On the other hand, most low-grade lymphomas have indolent behavior and sometimes do not need aggressive treatment [4]. Low-grade or early-stage lymphoma is quite challenged to differentiate from benign follicular hyperplasia, and additional diagnostic tools such as a clonality test or specific tumor biomarker are required. For the past decade, proteomics have been used to investigate lymphoma-specific proteins in several studies [5-8]. Two mass spectral peaks of m/z values 7041 and 74726 [8] and three mass spectral peaks of m/z 9242, 9452, and 9580 [6] were identified with discrimination between lymphoma and non-lymphoma proteomic serum profiles using surface-enhanced laser desorption ionization-time of flight (SELDI-TOF) mass spectrometry (MS). In another study, haptoglobin (Hp), α2 macroglobulin, α-antichymotrypsin, inter-α-trypsin inhibitor, and clusterin were identified in dog sera with a lymphoma versus a non-lymphoma group by two-dimensional electrophoresis (2DE) and tandem MS [5]. In addition, protein profiles in the lymph nodes have been reported in B-cell lymphoma and non-lymphoma dogs using 2DE and matrix-assisted laser desorption ionization (MALDI)-TOF MS [7]. The application of glycoproteomic techniques has also been studied the fucosylated peptide levels in sera of B-cell lymphoma dogs in one study [9]. Due to different proteomic techniques, diverse lymphoma subtypes, and dissimilar sample sizes of lymphoma and control groups, various proteins associated with lymphoma have been pro­posed; therefore determination of the diagnostic value of each protein for early diagnosis and prediction of progressive disease needs further investigation.

Another proteomic tool for quantitation of protein level in a sample is labeling isobaric tags, a high-resolution gel-free technique based on the mass difference between differentially labeled peptides. Nowadays, multiplexed sets of reagents are commercially available, for example, the isobaric tag for relative and absolute quantitation [10] and the tandem mass tag (TMT) [11]. Peptide labeling with isobaric mass tags enables precisely multiplexed peptide and protein quantification [12]. A TMT-proteomics approach is used to study protein profiles in human colon cancer [13], hepatocellular carcinoma [14], and lung adenocarcinoma [15]. This technique has been used to identify novel biomarkers in canine leishmaniosis and canine chronic valve disease [16,17]. However, no study has used this proteomics approach in canine lymphomas.

Thus, this study aimed to investigate and compare the differential sera proteomes between canine B-cell and T-cell lymphomas apart from healthy dogs by TMT and liquid chromatography–MS (LC–MS/MS).

## Materials and Methods

### Ethical approval

This study was approved by the Animal Care and Use Committee of the Faculty of Veterinary Science, Chulalongkorn University, Thailand (No. 1731053).

### Study period and location

This study was conducted from January 2015 to December 2017 at the Department of Pathology, Faculty of Veterinary Science, Chulalongkorn University, Bangkok, Thailand.

### Animals

A 2 ml of whole blood was collected from cephalic or lateral saphenous vein and kept in a plain tube from 20 lymphomas and four healthy adult dogs (annual vaccinations and deworming) at Small Animal Teaching Hospital, Faculty of Veterinary Science, Chulalongkorn University. Serum sample was then separated by centrifuge at 3,300 ×g for 5 min and stored at −80°C until used for proteomic analysis. Before sample collection, each patient did not receive any anti-neoplastic drug and did not have a complication such as hyperproteinemia. All patients were routinely examined for complete blood count, liver enzymes (alanine aminotransferase and alkaline phosphatase), kidney profiles (blood urea nitrogen and creatinine), and blood parasites (thin blood smear and SNAP4Dx Plus Test; IDEXX Laboratories, Westbrook, ME, USA). Moreover, thoracic radiography, abdominal ultrasonography, and buffy coat smear were performed for the WHO clinical staging. All cases were diagnosed as lymphoma based on the cytopathology, histopathology, and/or clonality test. The histopathological classification was categorized according to the Revised European American Lymphoma (REAL)/WHO [2]. The immunohistochemistry against CD3 and Pax5 for T and B lymphocyte markers was performed based on the previous report [18].

### Sample preparation and digestion

Each sera sample was measured for protein concentration by a modified pierce bicinchoninic acid protein assay kit (Thermo Fisher Scientific, Rockford, IL, USA). A total protein of 500 μg from the individual sample was reduced and alkylated with Tris (2-carboxyethyl) phosphine and iodoacetamide, respectively. Each sample was incubated with six volumes of pre-chilled acetone at –20°C overnight. After centrifugation, the pellet was resuspended with 100 mM triethylammonium bicarbonate buffer and digested with trypsin at a ratio of 1:40 (w/w) at 37°C overnight. After digestion, amount of peptides was measured by a peptide quantitation fluorometric peptide assay (Thermo Fisher).

### Tandem Mas Tag (TMT) labeling

The tryptic peptides from each sample were labeled with TMT 10 plexTM Isobaric Label Reagent Set (Thermo Fisher) following the manufacturer’s protocols. Samples were separated into three 8-plex groups i.e., Group 1: six lymphomas and two control samples, Group 2: seven lymphomas and one control sample, and Group 3: seven lymphomas and one control sample. The 8-plex samples in each group were labeled with the following TMT reagents: 127N, 127C, 128N, 128C, 129N, 129C, 130N, and 130C. After labeling, the samples were incubated for 1 h at 24°C--26°C and quenched with 5% hydroxylamine (Sigma-Aldrich) for 15 min. Each labeled sample was combined and dried in a vacuum centrifugation. Then, the pooled labeled peptides were separated into eight fractions using the Pierce High pH Reversed-Phase Peptide Fractionation Kit (Thermo Fisher) with a minor modified instruction. Then, all fractions were dried in vacuo and dissolved in 0.1% formic acid (Sigma-Aldrich) before MS analysis.

### LC–MS/MS

Analysis of samples was performed on an EASY-nLC1000 system coupled to a Q-Exactive Orbitrap Plus mass spectrometer equipped with a nano-electrospray ion source (Thermo Fisher). The peptides were eluted with the following acetonitrile gradients in 0.1% formic acid: 5–8% for 3 min, 8–15% for 60 min, 15–40% for 23 min, 40–95% for 2 min, and 95% for 5 min at a flow rate of 300 nL/min. The MS methods included a full MS scan at a resolution of 70,000 followed by 10 data-dependent MS2 scans at a resolution of 35,000. The normalized collision energy of HCD fragmentation was set at 32%. An MS scan range of 200 to 2000 m/z was selected and precursor ions with unassigned charge states, a charge state of +1, or a charge state of greater than +8 were excluded. A dynamic exclusion of 30 s was used. Peaklist-generating software of Thermo Xcalibur 3.0.63 (August 27, 2013) was used.

### Protein identification

Quantitative TMT data were analyzed by Proteome Discoverer™ Software 2.1.1.21. (Thermo Fisher) The MS raw data files were searched against a composite database containing the forward and reversed peptide sequences of the Canis lupus familiaris Swiss-Prot Database (836 proteins) with a list of common protein contaminants (www.thegpm.org/crap/). The search parameters were set for the following fixed modifications: carbamidomethylation of cysteine (+57.02146 Da), as well as static mass shift for TMT tags at N-termini and lysine (+229.162932 Da). For variable modifications, the search parameters were set as follows: oxidation of methionine (+15.99491 Da) with a maximum of four modifications and two missed cleavages allowed per peptide. Parent and fragment monoisotopic mass errors were set at 10 ppm and 0.02 Da, respectively. A target–decoy approach was used to limit a false discovery rate of the identified peptides to less than 1%. The reporter ion intensity ratios were transformed to log 2 ratios. P-values were calculated with Student’s t-test based on the triplicate log 2 ratios. The proteins with p-value≤ 0.05 were considered as significantly altered proteins.

### Statistical analysis

Differential protein expression was calculated and compared between lymphoma and healthy groups using the Mann–Whitney U-test, and among B-cell, T-cell lymphoma, and control dogs using the Kruskal–Wallis test. Statistical analysis was performed using GraphPad Prism version 9.3.0 for macOS (GraphPad Software, San Diego, CA, USA). P≤0.05 was considered statistically significant.

## Results

### Animals and lymphoma classification

The demographic data of 24 dogs are shown in Table-1. The control group consisted of four healthy dogs with normal blood profiles, aged from 3 to 9 years (mean 4.5 years old), of the following breeds: One American pit bull, one Labrador retriever, one Shih Tzu, and one mixed breed. In the lymphoma group, 20 dogs were aged from 2.3 to 15.7 years (mean 9.85 years old). The purebreds consisted of a Golden retriever, Shih Tzu, Saint Bernard, Pit Bull, American cocker spaniel, Thai Ridgeback, Italian greyhound, German shepherd, American bully, Spitz, Pomeranian, and Labrador retriever. For anatomical form, 16 cases were multicentric, three were mediastinal, and one was cutaneous lymphoma. Based on the immunophenotyping, 14 dogs were B-cell lymphomas (Pax5+, CD3-) and six dogs were T-cell lymphomas (Pax5-, CD3+). A clonality test was performed in four cases (mediastinal and cutaneous forms) and all samples showed a distinct peak of T-cell receptor gamma gene.

**Table 1:** Demographic and histopathological characteristics of 24 dogs in this study.

Study group	Variables	Number
B-cell lymphoma (14)	Mean age (range in years)	9.52 (2.3-14 years)
	Sex	
	Male	5
	Female	9
	Breed	
	Purebred	11
	Mixed	3
	WHO clinical stage	
	III	8
	IV	4
	V	2
	Sub-stage	
	A	9
	B	5
	REAL/WHO classification	
	Small lymphocytic B-cell lymphoma	2
	Lymphoplasmacytic B-cell lymphoma	1
	T-cell-rich B-cell lymphoma	3
	Diffuse large B-cell lymphoma	8
T-cell lymphoma (6)	Mean age (range in years)	10.62 (6.6-15.7 years)
	Sex	
	Male	4
	Female	2
	Breed	
	Purebred	6
	WHO clinical stage	
	III	2
	IV	4
	Sub-stage	
	A	1
	B	5
	REAL/WHO classification	
	Mediastinal T-cell lymphoma	3
	Epitheliotropic T-cell lymphoma	1
	Lymphoblastic T-cell lymphoma	1
	Peripheral T-cell lymphoma	1
Healthy dogs (4)	Mean age (range in years)	4.5 (3-9 years)
	Sex	
	Castrated male	2
	Sprayed female	2
	Breed	
	Purebred	3
	Mixed	1

REAL=Revised European American lymphoma, WHO=World Health Organization

### TMT labeling and LC–MS/MS

**Figure-1:**
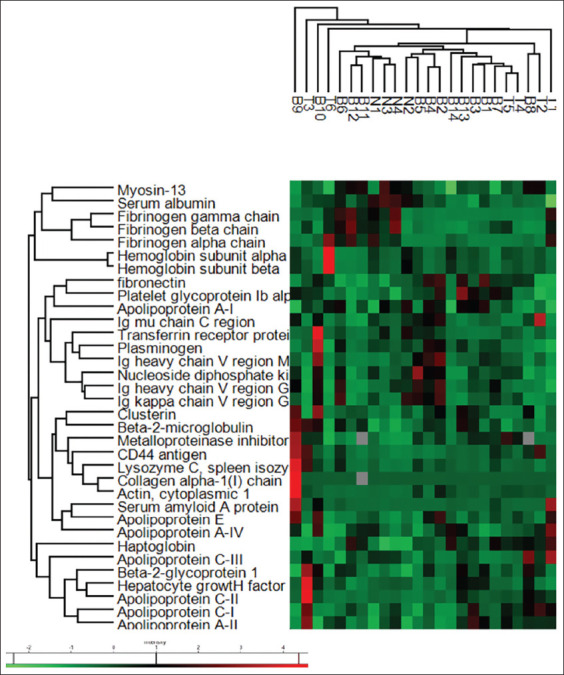
Heatmap visualization of protein intensities in 20 lymphomas and four healthy dogs. The column represents the lymphoma and normal sera samples. The rows represent the 34 differentially expressed proteins. Red color indicates a high expression level and green color indicates a low expression level.

We identified 34 individual proteins from *Canis lupus familiaris*, as shown in Figure-1. Among the 34 proteins, 20 proteins were upregulated and 14 proteins were downregulated expression in dogs with lymphoma (Table-2). Differently higher serum proteins in canine lymphoma were observed in beta-actin cytoplasmic 1 (ACTB, p=0.04), Hp (p=0.002), beta-2 microglobulin (β2M, p=0.007), beta-2 glycoprotein 1 (APOH, p=0.03), metalloproteinase inhibitor 1 (TIMP-1, p=0.03), and CD44 antigen (p=0.02). Serum albumin was significantly lower in lymphoma than in control dogs (p=0.0002).

**Table 2:** The 34 proteins with the highest (red color) and lowest (green color) relative expression in lymphoma and healthy control dogs.

Proteins	UniProt No.	Fold change (lymphoma/healthy)	p-value	Fold change (B/healthy)	Fold change (T/healthy)	Fold change (B/T)	p-value
Collagen alpha-1(I) chain	Q9XSJ7	13.55	0.77	19.00	1.74	10.92	0.23
Serum amyloid A protein	P19708	11.13	0.11	10.11	13.50	0.75	0.26
Haptoglobin	P19006	3.82	0.002*	3.81	3.84	0.99	0.02*
Lysozyme C, spleen isozyme	P81709	2.70	0.06	2.88	2.29	1.26	0.07
Beta-actin cytoplasmic 1	O18840	2.27	0.04*	2.49	1.76	1.42	0.10
Beta-2-microglobulin	P19341	2.10	0.007*	2.32	1.59	1.46	0.006*
Apolipoprotein C-II	P12278	1.99	0.24	1.48	3.16	0.47	0.13
CD44 antigen	Q28284	1.86	0.02*	1.71	2.19	0.78	0.07
Metalloproteinase inhibitor 1	P81546	1.86	0.03*	1.91	1.77	1.08	0.08
Ig heavy chain V region MOO	P01785	1.61	0.48	1.91	0.90	2.11	0.15
Apolipoprotein E	P18649	1.58	0.35	1.67	1.36	1.23	0.38
Apolipoprotein A-II	E2RAK7	1.48	0.35	1.27	1.95	0.65	0.13
Platelet glycoprotein Ib alpha chain	Q28256	1.47	0.31	1.64	1.07	1.53	0.12
Beta-2-glycoprotein 1	P33703	1.46	0.03*	1.45	1.47	0.99	0.08
Apolipoprotein C-I	P56595	1.40	0.52	1.13	2.03	0.56	0.05*
Fibronectin	Q28275	1.32	0.25	1.40	1.12	1.26	0.29
Clusterin	P25473	1.28	0.38	1.44	0.91	1.59	0.01*
Apolipoprotein A-IV	E2RE76	1.12	0.97	1.12	1.11	1.01	0.91
Hepatocyte growth factor activator	Q6QNF4	1.12	0.42	1.08	1.20	0.91	0.37
Ig heavy chain V region GOM	P01784	1.03	0.68	1.24	0.55	2.27	0.02*
Ig mu chain C region	P01874	0.98	0.48	1.00	0.93	1.07	0.23
Plasminogen	P80009	0.97	0.35	1.09	0.70	1.56	0.02*
Nucleoside diphosphate kinase A	Q50KA9	0.95	0.52	1.09	0.63	1.74	0.17
Apolipoprotein C-III	P12279	0.92	0.16	0.81	1.16	0.70	0.07
Ig kappa chain V region GOM	P01618	0.92	0.53	1.05	0.61	1.72	0.17
Apolipoprotein A-I	P02648	0.91	0.52	0.93	0.85	1.10	0.64
Hemoglobin subunit alpha	P60529	0.87	0.31	0.65	1.39	0.47	0.27
Myosin-13	Q076A3	0.86	0.21	0.86	0.86	1.01	0.42
Hemoglobin subunit beta	P60524	0.84	0.39	0.60	1.42	0.42	0.21
Serum albumin	P49822	0.78	0.0002*	0.79	0.74	1.07	0.007*
Transferrin receptor protein 1	Q9GLD3	0.76	0.08	0.81	0.64	1.26	0.20
Fibrinogen alpha chain	P68213	0.62	0.52	0.49	0.94	0.52	0.63
Fibrinogen beta chain	P02677	0.58	0.39	0.54	0.67	0.81	0.44
Fibrinogen gamma chain	P12800	0.49	0.39	0.46	0.55	0.84	0.54

Red font color=Protein upregulation, Green font color=Protein downregulation,

* Bold font=Statistical difference

When we compared three groups of B-cell lymphoma, T-cell lymphoma, and healthy dogs, the serum β2M (p=0.006), clusterin (p=0.01), immunoglobulin (Ig) heavy chain V region GOM (p=0.02), and plasminogen (p=0.02) were significantly higher in B-cell than T-cell lymphoma. In T-cell lymphoma, serum apolipoprotein C1 (APOC1, p=0.05) was elevated compared to B-cell lymphoma and healthy dogs. Serum Hp was significantly increased in either B-cell or T-cell lymphoma compared to the control (p=0.02). Serum albumin was significantly lower in both T-cell and B-cell lymphomas than healthy controls (p=0.007).

The variable factors of clinical stage, sub-stage, and immunophenotype were selected to calculate their effects on the protein levels of albumin, ACTB, β2M, APOH, TIMP-1, CD44, Ig heavy chain V region GOM, APOC1, clusterin, and plasminogen through the least-squares for multiple regression method. Clinical stage influenced the differential expression of ACTB (p=0.0001), TIMP-1 (p=0.02), Hp (p=0.02), β2M (p=0.01), and albumin (p=0.0006). The sub-stage was involved with the Hp (p=0.02) and albumin (p=0.0001) quantities. The immunophenotype impacted the quantities of Hp (p=0.03), β2M (p=0.02), clusterin (p=0.01), Ig heavy chain V region GOM (p=0.02), APOC1 (p=0.02), and albumin (p<0.0001).

## Discussion

The authors used a TMT-based technique and LC–MS/MS to detect the quantitative serum proteomic profiles in B-cell lymphomas, T-cell lymphomas, and healthy dogs. In neoplastic B phenotypes, drastically differential increased protein levels were observed in Ig heavy chain V region GOM, clusterin, β2M, and plasminogen, while APOC1 was only notably elevated in T-cell lymphoma. TMT could quantify multiplexed peptides and label proteins, which are easily achieved through automation. TMT provides higher accuracy than other labeling-based methods, including gel-based techniques with low throughput and poor dynamic range [19].

The first reported serum proteins in canine lymphoma were ACTB, Ig heavy chain V region GOM, TIMP-1, APOH, CD44, APOC1, and β2M. Actin cytoplasmic 1 or beta-actin (ACTB) is highly conserved in cytoskeletal structural proteins in all eukaryotic cells. The abnormal expression and polymerization of ACTB have been associated with cancer invasiveness and metastasis. A high abundance of ACTB was correlated with cell proliferation of breast cancer cell lines in one study [20]. Ig heavy chain V region GOM serves as an antigen-binding site and is involved in the B-cell receptor signaling pathway. No study has previously reported the increased expression of Ig heavy chain V region GOM associated with canine lymphoma in the same way as with TIMP-1, APOH, and CD44. TIMP-1 is one of the inhibitors of matrix metalloproteinase. Serum TIMP-1 plays a potential role in the diagnosis and prognosis of human cancers, including gastric and colorectal cancers [21,22]. APOH is affected to angiogenesis and endothelial cell growth. This protein was proposed as serum biomarker for human hepatocellular carcinoma [23]. Elevated serum CD44, a transmembrane glycoprotein involving lymphocyte activation, adhesion, and migration, was correlated with the prognosis outcome in human non-Hodgkin lymphoma [24,25]. Therefore, the significance of these proteins in the lymphoma pathogenesis and the valuable tool for diagnosis and prognosis need further investigation in dogs.

APOC1, the smallest member of the apolipoprotein family, is secreted by the liver and forms a component of both triglyceride-rich lipoproteins and high-density lipoproteins. After apolipoprotein binds to a lipid and forms lipoprotein, it transports the lipid into the lymphatic and circulatory systems. APOC1 and other apolipoprotein subclasses regulate and control serum and plasma lipoprotein metabolism. Recent studies in humans have shown that APOC1 may associate with the development of cancers. Elevated serum APOC1 was observed in patients with pancreatic cancer [26], gastric cancer [27], and triple-negative breast cancer [28]. It also correlated with decreased overall survival. Takano *et al*. [26] suggested that pancreatic neoplastic cells secreted APOC1, which prevented spontaneous apoptosis. However, there is no evidence suggesting its role and prognostic value in canine lymphomas.

β2M is synthesized in all nucleated cells and forms the light chain subunit of the major histocompatibility complex Class I antigen. Soluble β2M can be detected in most biological fluids, such as blood, urine, and cerebrospinal fluid, after release from the intracellular or cell surface. In cancers, β2M is involved in the regulation of cell survival, proliferation, apoptosis, and metastasis [29,30]. Elevated serum β2M is reported in human cancers, including many lymphoma subtypes. The level of β2M served as a prognostic biomarker, which was strongly associated with poor overall survival in patients with diffuse large B-cell lymphoma [31], mantle cell lymphoma [32], follicular lymphoma [33], Hodgkin lymphoma [34], and angioimmunoblastic T-cell lymphoma [35]. In one study, the concentration of β2M in a urine sample could indicate an early to mid-stage of tubular injury in dogs [36]. However, no study has reported on β2M upregulation in serum or its significant prognosis in canine lymphomas.

This study observed high serum clusterin or apolipoprotein J in canine lymphoma, especially the B-cell subtype. Clusterin is a secreted heterodimeric glycoprotein that is widely distributed in human tissues and fluids [37]. This protein plays several biological roles in tumorigenesis, which include apoptosis, cell cycle regulation, and immune system regulation [38,39]. A recent study evaluated the serum clusterin levels in canine multicentric lymphoma and found that dogs with lymphoma had lower clusterin levels than healthy dogs [40], dissimilar to our results. In humans, clusterin expression was specific to anaplastic large-cell lymphoma and provided an additional diagnostic marker [41]. Its increased expression in human cutaneous T-cell lymphoma was related to an unfavorable prognosis [42]. Consequently, the role of clusterin in specific lymphoma subtypes in dogs needs to confirm in future studies.

In addition, acute-phase proteins are secreted into the bloodstream in response to any tissue injury. During the acute-phase response, some proteins increase in concentration (positive acute-phase proteins, e.g., C-reactive protein, Hp, and fibrinogen), while others decrease (negative acute-phase proteins, e.g., albumin or transferrin). In dogs, Hp is a moderate acute-phase protein that is heightened by approximately 2-3 times, and albumin is a negative acute-phase protein that reduces during inflammation [43]. Elevated serum Hp concentrations and low concentrations of serum albumin was observed in our study, similar to the previous reports of canine high-grade multicentric lymphomas [5,44]. The canine lymphoma blood test values, derived from C-reactive protein and Hp, could predict the remission status and relate to the survival time [45]. In addition, hypoalbuminemia was significantly associated with reduced survival time and progression-free survival in canine lymphomas [46]. As such, the serum levels of Hp and albumin are useful for the prognosis in dogs with lymphoma.

The major limitation of this study was the small sample sizes in both lymphoma subtype and control group. Moreover, four healthy adult dogs had various ages, breeds, and sexes that could lead to great variability in their serum proteomic profiles. When compared between B-cell and T-cell lymphoma, each case had different clinical stages, anatomic forms, and histopathological subtypes. Thus, it was challenging to discover a specific protein in each subgroup. For serum proteomic analysis, the authors did not perform immune-affinity depletion to eliminate the most abundant proteins, such as albumin, which might be interfered with other peptides’ measurement.

## Conclusion

Our study evaluated the serum proteome in canine B- and T-cell lymphomas using TMT and LC–MS/MS. Potential novel candidate biomarkers in canine lymphoma were ACTB, β2M, TIMP-1, CD44 antigen, Ig heavy chain V region GOM, APOC1, and APOH. Further investigation of these serum proteins is needed to confirm their roles in lymphomagenesis and diagnostic and prognostic capability in specific lymphoma subtypes of this species.

## Data Availability

Supplementary data can be available from the corresponding author on reasonable request.

## Authors’ Contributions

PF, KR, SS, and AR: Contributed to the conception, designed the study, and analyzed the data. TP: Contributed to the supervision. PF, SS, and AR: Drafted and revised the manuscript. All authors have read and approved the final manuscript.
